# 1-(4-Methoxy­phen­yl)-7-phenyl-3-(phenyl­selenylmeth­yl)perhydro­isoxazolo[2′,3′:1,2]pyrrolo[3,4-*b*]azetidine-6-spiro-2′-chroman-2,4′-dione

**DOI:** 10.1107/S1600536808008829

**Published:** 2008-04-16

**Authors:** E. Theboral Sugi Kamala, S. Nirmala, L. Sudha, N. Arumugam, R. Raghunathan

**Affiliations:** a Department of Physics, Easwari Engineering College, Ramapuram, Chennai 600 089, India; b Department of Physics, SRM University, Ramapuram Campus, Chennai 600 089, India; c Department of Organic Chemistry, University of Madras, Guindy Campus, Chennai 600 025, India

## Abstract

In the title compound, C_35_H_30_N_2_O_5_Se, the pyrrolidine ring adopts an envelope conformation and the oxazolidine ring is in a twist conformation. The tetra­hydro­pyran ring adopts a half-chair conformation. The methoxy­phenyl ring is twisted away from the attached azetidinone ring by 15.7 (1)°. In the crystal structure, inter­molecular C—H⋯O inter­actions link the mol­ecules into a two-dimensional network.

## Related literature

For general background, see: Brakhage (1998 ▶); Chenera *et al.* (1993 ▶); Ellis (1997 ▶); Farrugia (1997 ▶); Kilonda *et al.* (1995 ▶); Koojiman *et al.* (1984 ▶); Lampronti *et al.* (2003 ▶). For bond-length data, see: Allen *et al.* (1987 ▶). For ring conformation details, see: Cremer & Pople (1975 ▶); Nardelli (1983 ▶).
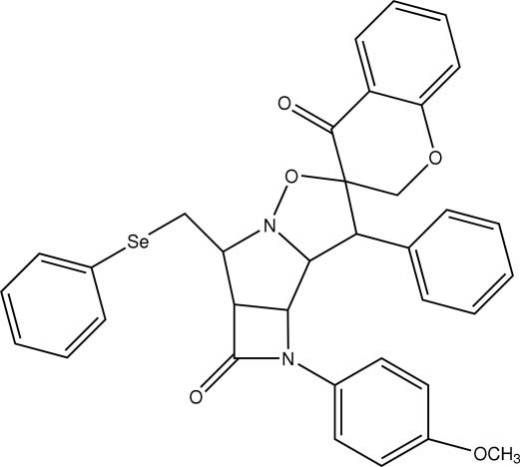


## Experimental

### 

#### Crystal data

C_35_H_30_N_2_O_5_Se
*M* *_r_* = 637.57
Monoclinic, 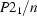

*a* = 14.0886 (5) Å
*b* = 10.6813 (4) Å
*c* = 19.4744 (7) Å
β = 92.721 (2)°
*V* = 2927.29 (18) Å^3^
*Z* = 4
Mo *K*α radiation
μ = 1.33 mm^−1^
*T* = 293 (2) K
0.30 × 0.20 × 0.20 mm

#### Data collection

Bruker Kappa APEXII diffractometer
Absorption correction: multi-scan (Blessing, 1995 ▶) *T*_min_ = 0.691, *T*_max_ = 0.777
38750 measured reflections
9941 independent reflections
5805 reflections with *I* > 2σ(*I*)
*R*_int_ = 0.028

#### Refinement

*R*[*F* ^2^ > 2σ(*F* ^2^)] = 0.045
*wR*(*F* ^2^) = 0.116
*S* = 1.01
9941 reflections
389 parameters
H-atom parameters constrained
Δρ_max_ = 0.79 e Å^−3^
Δρ_min_ = −0.67 e Å^−3^

### 

Data collection: *APEX2* (Bruker, 2004 ▶); cell refinement: *APEX2*; data reduction: *SAINT* (Bruker, 2004 ▶); program(s) used to solve structure: *SHELXS97* (Sheldrick, 2008 ▶); program(s) used to refine structure: *SHELXL97* (Sheldrick, 2008 ▶); molecular graphics: *ORTEP-3* (Farrugia, 1997 ▶); software used to prepare material for publication: *PLATON* (Spek, 2003 ▶).

## Supplementary Material

Crystal structure: contains datablocks I, global. DOI: 10.1107/S1600536808008829/ci2571sup1.cif

Structure factors: contains datablocks I. DOI: 10.1107/S1600536808008829/ci2571Isup2.hkl

Additional supplementary materials:  crystallographic information; 3D view; checkCIF report

## Figures and Tables

**Table 1:** Hydrogen-bond geometry (Å, °)

*D*—H⋯*A*	*D*—H	H⋯*A*	*D*⋯*A*	*D*—H⋯*A*
C5—H5⋯O1^i^	0.98	2.42	3.199 (3)	136
C29—H29*A*⋯O5^ii^	0.97	2.54	3.465 (3)	160

Symmetry codes: (i) 
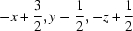
; (ii) 
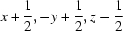
.

**Fig. 1.**
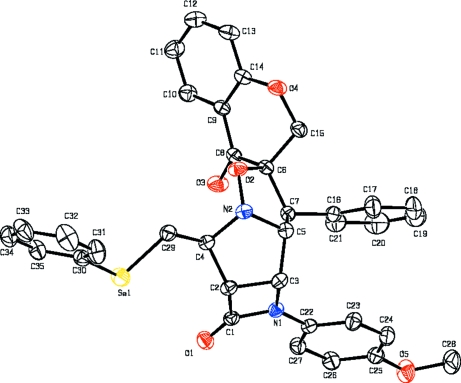
The molecular structure of the title compound with 30% probability displacement ellipsoids.

**Fig. 2.**
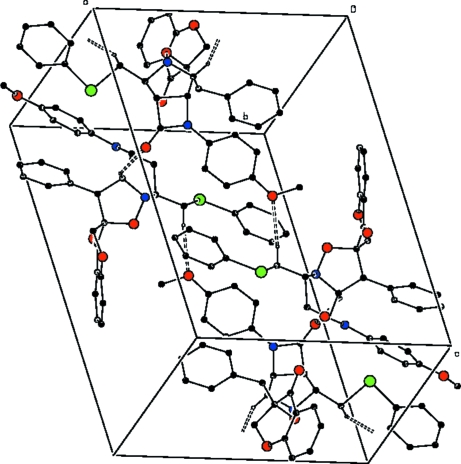
A view of the molecular packing of the title compound.

## supplementary crystallographic information

### Comment

Chromanones are found to exhibit strong activity in inhibiting *in vitro* cell growth of human tumour cells (Lampronti *et al.*, 2003). Many chromanone derivatives are versatile intermediates for the synthesis of natural products such as brazillin, hematoxylin, ripariochromene, clausenin, calonlide A and inophyllum B (Koojiman *et al.*,1984; Ellis *et al.*, 1997; Chenera *et al.*, 1993). Pyrrolidines and pyrroles are common structural motifs in drugs and drug candidates owing to their ability to act as selective glycosidase inhibitors which are used in the treatment of diabetes, cancer, malaria and viral infections including AIDS (Kilonda *et al.*,1995). The most commonly used β-lactam antibiotics for the therapy of infectious diseases are penicillin and cephalosporin (Brakhage, 1998). In view of the above, the crystal structure determination of the title compound was carried out and the results are presented here.

Bond lengths of the title compound (Fig. 1) show normal values (Allen *et al.*, 1987). The pyrrolidine ring (N2/C4/C2/C3/C5) adopts an envelope conformation with an asymmetry parameter (Nardelli,1983) ΔC_s_(N2) of 3.8 (2)° and puckering parameters (Cremer and Pople, 1975) of q_2_ = 0.366 (2) Å and φ_2_ = 184.9 (3)°. The oxazolidine ring (O2/N2/C5/C7/C6) is in a twist conformation with an asymmetry parameter ΔC_2_(O2) of 8.9 (2)° and puckering parameters q2 = 0.329 (2) Å and φ2 = 262.2 (3)°. The sums of bond angles around atom N1 (359.7°) and N2 (324.5°) indicate *sp*^2^ and *sp*^3^ hybridization, respectively. The tetrahydropyran ring adopts a half-chair conformation. The dihedral angle between the azetidinone ring and the attached methoxyphenyl ring is 15.7 (1)°.

In the crystal packing, intermolecular C—H···O interactions (Table 1) link the molecules into a two-dimensional network (Fig. 2).

### Experimental

To a solution of the bicyclic nitrone (1 mol) in dry acetonitrile (20 ml) was added 3-arylidene chromanone (1 mol) under a N_2_ atmosphere. The reaction was refluxed for 4 h. After the completion of the reaction, the solvent was distilled off under reduced pressure and the crude product was purified by column chromatography. The title compound was crystallized from benzene solution by slow evaporation technique.

### Refinement

H atoms were placed in idealized positions and allowed to ride on their parent atoms, with C-H = 0.93-0.98 Å and *U*_iso_(H)= 1.2–1.5*U*_eq_(C).

### Crystal data

**Table:**

C_35_H_30_N_2_O_5_Se	*F*_000_ = 1312
*M**_r_* = 637.57	*D*_x_ = 1.447 Mg m^−^^3^
Monoclinic, *P*2_1_/*n*	Mo *K*α radiation λ = 0.71073 Å
Hall symbol: -P 2yn	Cell parameters from 38750 reflections
*a* = 14.0886 (5) Å	θ = 1.8–31.9º
*b* = 10.6813 (4) Å	µ = 1.33 mm^−^^1^
*c* = 19.4744 (7) Å	*T* = 293 (2) K
β = 92.721 (2)º	Prism, colourless
*V* = 2927.29 (18) Å^3^	0.30 × 0.20 × 0.20 mm
*Z* = 4	

### Data collection

**Table:**

Bruker KappaAPEX2 diffractometer	9941 independent reflections
Radiation source: fine-focus sealed tube	5805 reflections with *I* > 2σ(*I*)
Monochromator: graphite	*R*_int_ = 0.028
*T* = 293(2) K	θ_max_ = 31.9º
ω and φ scans	θ_min_ = 1.8º
Absorption correction: multi-scan(Blessing, 1995)	*h* = −19→20
*T*_min_ = 0.691, *T*_max_ = 0.777	*k* = −15→10
38750 measured reflections	*l* = −28→28

### Refinement

**Table:**

Refinement on *F*^2^	Secondary atom site location: difference Fourier map
Least-squares matrix: full	Hydrogen site location: inferred from neighbouring sites
*R*[*F*^2^ > 2σ(*F*^2^)] = 0.045	H-atom parameters constrained
*wR*(*F*^2^) = 0.116	*w* = 1/[σ^2^(*F*_o_^2^) + (0.0444*P*)^2^ + 1.2941*P*] where *P* = (*F*_o_^2^ + 2*F*_c_^2^)/3
*S* = 1.01	(Δ/σ)_max_ = 0.001
9941 reflections	Δρ_max_ = 0.79 e Å^−^^3^
389 parameters	Δρ_min_ = −0.67 e Å^−^^3^
Primary atom site location: structure-invariant direct methods	Extinction correction: none

### Special details

**Table:**

Geometry. All e.s.d.'s (except the e.s.d. in the dihedral angle between two l.s. planes) are estimated using the full covariance matrix. The cell e.s.d.'s are taken into account individually in the estimation of e.s.d.'s in distances, angles and torsion angles; correlations between e.s.d.'s in cell parameters are only used when they are defined by crystal symmetry. An approximate (isotropic) treatment of cell e.s.d.'s is used for estimating e.s.d.'s involving l.s. planes.
Refinement. Refinement of *F*^2^ against ALL reflections. The weighted *R*-factor *wR* and goodness of fit *S* are based on *F*^2^, conventional *R*-factors *R* are based on *F*, with *F* set to zero for negative *F*^2^. The threshold expression of *F*^2^ > σ(*F*^2^) is used only for calculating *R*-factors(gt) *etc*. and is not relevant to the choice of reflections for refinement. *R*-factors based on *F*^2^ are statistically about twice as large as those based on *F*, and *R*- factors based on ALL data will be even larger.

### Fractional atomic coordinates and isotropic or equivalent isotropic displacement parameters (Å^2^)

**Table:**

	*x*	*y*	*z*	*U*_iso_*/*U*_eq_	
Se1	1.016999 (16)	0.19600 (3)	0.180072 (11)	0.05427 (9)	
O1	0.84548 (10)	0.24863 (15)	0.33002 (7)	0.0499 (4)	
O2	0.69775 (9)	0.13919 (14)	0.07844 (6)	0.0423 (3)	
O3	0.64805 (13)	0.40844 (15)	0.12826 (7)	0.0583 (4)	
O4	0.54329 (11)	0.16190 (14)	−0.01690 (7)	0.0482 (4)	
O5	0.42581 (12)	0.20236 (17)	0.48343 (9)	0.0662 (5)	
N1	0.71402 (11)	0.11790 (15)	0.30571 (7)	0.0353 (3)	
N2	0.74055 (11)	0.06570 (15)	0.13393 (7)	0.0358 (3)	
C1	0.80284 (13)	0.16622 (18)	0.30008 (9)	0.0359 (4)	
C2	0.82270 (13)	0.07814 (18)	0.24043 (9)	0.0357 (4)	
H2	0.8749	0.0189	0.2497	0.043*	
C3	0.72246 (13)	0.02471 (18)	0.25094 (9)	0.0354 (4)	
H3	0.7221	−0.0623	0.2669	0.042*	
C4	0.81961 (13)	0.13643 (18)	0.16898 (9)	0.0343 (4)	
H4	0.8039	0.2256	0.1714	0.041*	
C5	0.66549 (13)	0.04692 (18)	0.18298 (9)	0.0346 (4)	
H5	0.6280	−0.0276	0.1704	0.041*	
C6	0.60958 (12)	0.19551 (17)	0.09998 (9)	0.0322 (4)	
C7	0.60343 (12)	0.16392 (17)	0.17651 (9)	0.0324 (4)	
H7	0.6359	0.2308	0.2028	0.039*	
C8	0.62051 (13)	0.33430 (18)	0.08468 (9)	0.0352 (4)	
C9	0.60212 (13)	0.36900 (18)	0.01199 (9)	0.0357 (4)	
C10	0.62385 (15)	0.4882 (2)	−0.01152 (11)	0.0458 (5)	
H10	0.6456	0.5490	0.0195	0.055*	
C11	0.61339 (18)	0.5166 (2)	−0.08025 (12)	0.0552 (6)	
H11	0.6283	0.5963	−0.0957	0.066*	
C12	0.58075 (18)	0.4265 (2)	−0.12602 (11)	0.0580 (6)	
H12	0.5743	0.4459	−0.1726	0.070*	
C13	0.55774 (17)	0.3095 (2)	−0.10459 (11)	0.0525 (6)	
H13	0.5355	0.2498	−0.1362	0.063*	
C14	0.56779 (14)	0.27993 (19)	−0.03503 (10)	0.0396 (4)	
C15	0.52914 (14)	0.1432 (2)	0.05444 (10)	0.0421 (4)	
H15A	0.5232	0.0542	0.0633	0.051*	
H15B	0.4702	0.1830	0.0661	0.051*	
C16	0.50369 (13)	0.1537 (2)	0.20162 (10)	0.0412 (4)	
C17	0.45117 (18)	0.0450 (3)	0.19745 (14)	0.0660 (7)	
H17	0.4770	−0.0262	0.1781	0.079*	
C18	0.3608 (2)	0.0404 (4)	0.22162 (16)	0.0898 (11)	
H18	0.3265	−0.0339	0.2186	0.108*	
C19	0.3214 (2)	0.1434 (5)	0.24973 (16)	0.0944 (12)	
H19	0.2604	0.1396	0.2659	0.113*	
C20	0.3719 (2)	0.2522 (4)	0.25400 (15)	0.0848 (10)	
H20	0.3450	0.3229	0.2730	0.102*	
C21	0.46266 (16)	0.2584 (3)	0.23037 (12)	0.0561 (6)	
H21	0.4965	0.3331	0.2337	0.067*	
C22	0.64158 (13)	0.13740 (18)	0.35180 (9)	0.0355 (4)	
C23	0.57163 (15)	0.0489 (2)	0.35695 (10)	0.0455 (5)	
H23	0.5739	−0.0240	0.3309	0.055*	
C24	0.49788 (16)	0.0667 (2)	0.40035 (11)	0.0491 (5)	
H24	0.4507	0.0063	0.4035	0.059*	
C25	0.49483 (14)	0.1746 (2)	0.43884 (10)	0.0448 (5)	
C26	0.56613 (15)	0.2627 (2)	0.43419 (11)	0.0474 (5)	
H26	0.5647	0.3348	0.4608	0.057*	
C27	0.63907 (14)	0.2453 (2)	0.39089 (10)	0.0424 (4)	
H27	0.6864	0.3055	0.3879	0.051*	
C28	0.34568 (18)	0.1226 (3)	0.48395 (15)	0.0736 (8)	
H28A	0.3016	0.1544	0.5158	0.110*	
H28B	0.3655	0.0400	0.4977	0.110*	
H28C	0.3156	0.1194	0.4387	0.110*	
C29	0.90857 (14)	0.1174 (2)	0.13028 (10)	0.0441 (5)	
H29A	0.9003	0.1540	0.0848	0.053*	
H29B	0.9205	0.0286	0.1250	0.053*	
C30	1.11387 (14)	0.1421 (2)	0.12143 (10)	0.0423 (4)	
C31	1.12365 (18)	0.0189 (2)	0.10270 (15)	0.0628 (7)	
H31	1.0823	−0.0412	0.1187	0.075*	
C32	1.19504 (19)	−0.0161 (3)	0.05994 (17)	0.0766 (8)	
H32	1.2007	−0.0994	0.0468	0.092*	
C33	1.25653 (18)	0.0703 (3)	0.03718 (14)	0.0715 (8)	
H33	1.3050	0.0461	0.0092	0.086*	
C34	1.24733 (17)	0.1910 (3)	0.05514 (14)	0.0641 (7)	
H34	1.2893	0.2502	0.0391	0.077*	
C35	1.17647 (15)	0.2283 (2)	0.09712 (12)	0.0523 (5)	
H35	1.1710	0.3122	0.1090	0.063*	

### Atomic displacement parameters (Å^2^)

**Table:**

	*U*^11^	*U*^22^	*U*^33^	*U*^12^	*U*^13^	*U*^23^
Se1	0.04250 (13)	0.07341 (18)	0.04728 (13)	0.00234 (11)	0.00606 (9)	−0.01202 (11)
O1	0.0460 (8)	0.0529 (9)	0.0506 (8)	−0.0080 (7)	0.0006 (7)	−0.0064 (7)
O2	0.0403 (7)	0.0574 (9)	0.0294 (6)	0.0159 (6)	0.0042 (5)	−0.0001 (6)
O3	0.0896 (12)	0.0422 (9)	0.0416 (8)	−0.0170 (8)	−0.0122 (8)	−0.0078 (7)
O4	0.0623 (9)	0.0430 (8)	0.0380 (7)	−0.0094 (7)	−0.0108 (6)	−0.0066 (6)
O5	0.0488 (9)	0.0835 (13)	0.0684 (11)	−0.0043 (8)	0.0253 (8)	−0.0200 (9)
N1	0.0391 (8)	0.0353 (9)	0.0317 (7)	0.0000 (7)	0.0052 (6)	−0.0020 (6)
N2	0.0373 (8)	0.0384 (9)	0.0318 (7)	0.0091 (7)	0.0030 (6)	−0.0002 (6)
C1	0.0366 (10)	0.0386 (11)	0.0324 (8)	0.0039 (8)	0.0002 (7)	0.0068 (8)
C2	0.0367 (9)	0.0369 (11)	0.0337 (8)	0.0093 (8)	0.0030 (7)	0.0043 (8)
C3	0.0426 (10)	0.0296 (9)	0.0343 (8)	0.0053 (8)	0.0064 (7)	0.0023 (7)
C4	0.0346 (9)	0.0366 (10)	0.0320 (8)	0.0071 (8)	0.0037 (7)	0.0019 (7)
C5	0.0356 (9)	0.0316 (10)	0.0368 (9)	0.0018 (7)	0.0043 (7)	−0.0045 (7)
C6	0.0291 (8)	0.0354 (10)	0.0319 (8)	0.0033 (7)	−0.0003 (6)	−0.0067 (7)
C7	0.0290 (8)	0.0338 (10)	0.0346 (8)	0.0012 (7)	0.0026 (7)	−0.0051 (7)
C8	0.0329 (9)	0.0366 (10)	0.0359 (9)	−0.0005 (8)	−0.0018 (7)	−0.0051 (8)
C9	0.0328 (9)	0.0370 (11)	0.0370 (9)	0.0044 (8)	−0.0031 (7)	−0.0028 (8)
C10	0.0509 (12)	0.0403 (12)	0.0460 (11)	0.0000 (9)	−0.0015 (9)	−0.0025 (9)
C11	0.0664 (15)	0.0471 (13)	0.0518 (12)	0.0032 (11)	−0.0001 (11)	0.0106 (11)
C12	0.0684 (16)	0.0632 (16)	0.0415 (11)	0.0064 (13)	−0.0073 (10)	0.0058 (11)
C13	0.0604 (14)	0.0559 (15)	0.0397 (10)	0.0007 (11)	−0.0131 (10)	−0.0056 (10)
C14	0.0369 (10)	0.0424 (12)	0.0386 (9)	0.0031 (8)	−0.0077 (8)	−0.0047 (8)
C15	0.0442 (11)	0.0393 (11)	0.0422 (10)	−0.0072 (9)	−0.0047 (8)	−0.0056 (9)
C16	0.0327 (10)	0.0552 (13)	0.0360 (9)	0.0011 (9)	0.0044 (7)	−0.0014 (9)
C17	0.0502 (14)	0.0756 (18)	0.0733 (16)	−0.0166 (13)	0.0132 (12)	−0.0070 (14)
C18	0.0539 (17)	0.138 (3)	0.0782 (19)	−0.0374 (19)	0.0110 (15)	0.004 (2)
C19	0.0363 (14)	0.181 (4)	0.0671 (18)	0.004 (2)	0.0132 (12)	0.010 (2)
C20	0.0533 (17)	0.134 (3)	0.0680 (17)	0.0316 (19)	0.0163 (14)	−0.0062 (19)
C21	0.0462 (13)	0.0687 (16)	0.0539 (12)	0.0178 (11)	0.0093 (10)	−0.0033 (12)
C22	0.0382 (10)	0.0385 (11)	0.0299 (8)	0.0018 (8)	0.0021 (7)	0.0019 (8)
C23	0.0525 (12)	0.0419 (12)	0.0431 (10)	−0.0058 (9)	0.0124 (9)	−0.0067 (9)
C24	0.0471 (12)	0.0500 (13)	0.0512 (12)	−0.0109 (10)	0.0115 (9)	−0.0024 (10)
C25	0.0365 (10)	0.0601 (14)	0.0384 (10)	0.0029 (9)	0.0075 (8)	−0.0040 (9)
C26	0.0467 (12)	0.0502 (13)	0.0457 (11)	−0.0004 (10)	0.0073 (9)	−0.0151 (10)
C27	0.0419 (11)	0.0432 (11)	0.0424 (10)	−0.0050 (9)	0.0049 (8)	−0.0083 (9)
C28	0.0448 (14)	0.099 (2)	0.0793 (18)	−0.0042 (14)	0.0260 (12)	−0.0061 (16)
C29	0.0366 (10)	0.0604 (14)	0.0359 (9)	0.0025 (9)	0.0062 (8)	−0.0018 (9)
C30	0.0331 (10)	0.0500 (12)	0.0437 (10)	0.0037 (9)	−0.0003 (8)	−0.0039 (9)
C31	0.0492 (13)	0.0477 (14)	0.0919 (19)	−0.0018 (11)	0.0087 (13)	−0.0055 (13)
C32	0.0586 (16)	0.0615 (17)	0.110 (2)	0.0086 (13)	0.0066 (15)	−0.0344 (16)
C33	0.0411 (13)	0.099 (2)	0.0751 (17)	−0.0032 (14)	0.0079 (12)	−0.0348 (16)
C34	0.0431 (13)	0.085 (2)	0.0653 (15)	−0.0131 (12)	0.0090 (11)	−0.0100 (14)
C35	0.0420 (12)	0.0548 (14)	0.0601 (13)	−0.0032 (10)	0.0028 (10)	−0.0081 (11)

### Geometric parameters (Å, °)

**Table:**

Se1—C30	1.910 (2)	C13—H13	0.93
Se1—C29	1.959 (2)	C15—H15A	0.97
O1—C1	1.201 (2)	C15—H15B	0.97
O2—N2	1.4441 (19)	C16—C17	1.377 (3)
O2—C6	1.460 (2)	C16—C21	1.389 (3)
O3—C8	1.211 (2)	C17—C18	1.379 (4)
O4—C14	1.358 (2)	C17—H17	0.93
O4—C15	1.427 (2)	C18—C19	1.359 (5)
O5—C25	1.367 (2)	C18—H18	0.93
O5—C28	1.414 (3)	C19—C20	1.364 (5)
N1—C1	1.363 (2)	C19—H19	0.93
N1—C22	1.406 (2)	C20—C21	1.381 (4)
N1—C3	1.468 (2)	C20—H20	0.93
N2—C5	1.472 (2)	C21—H21	0.93
N2—C4	1.485 (2)	C22—C23	1.373 (3)
C1—C2	1.531 (3)	C22—C27	1.382 (3)
C2—C4	1.523 (2)	C23—C24	1.383 (3)
C2—C3	1.546 (3)	C23—H23	0.93
C2—H2	0.98	C24—C25	1.376 (3)
C3—C5	1.533 (3)	C24—H24	0.93
C3—H3	0.98	C25—C26	1.382 (3)
C4—C29	1.506 (3)	C26—C27	1.372 (3)
C4—H4	0.98	C26—H26	0.93
C5—C7	1.527 (3)	C27—H27	0.93
C5—H5	0.98	C28—H28A	0.96
C6—C15	1.513 (2)	C28—H28B	0.96
C6—C8	1.521 (3)	C28—H28C	0.96
C6—C7	1.535 (2)	C29—H29A	0.97
C7—C16	1.513 (2)	C29—H29B	0.97
C7—H7	0.98	C30—C31	1.374 (3)
C8—C9	1.474 (3)	C30—C35	1.375 (3)
C9—C14	1.391 (3)	C31—C32	1.387 (4)
C9—C10	1.392 (3)	C31—H31	0.93
C10—C11	1.374 (3)	C32—C33	1.354 (4)
C10—H10	0.93	C32—H32	0.93
C11—C12	1.376 (3)	C33—C34	1.344 (4)
C11—H11	0.93	C33—H33	0.93
C12—C13	1.362 (3)	C34—C35	1.379 (3)
C12—H12	0.93	C34—H34	0.93
C13—C14	1.392 (3)	C35—H35	0.93
			
C30—Se1—C29	97.90 (8)	O4—C15—H15A	109.1
N2—O2—C6	109.86 (12)	C6—C15—H15A	109.1
C14—O4—C15	115.61 (15)	O4—C15—H15B	109.1
C25—O5—C28	117.78 (19)	C6—C15—H15B	109.1
C1—N1—C22	133.82 (16)	H15A—C15—H15B	107.8
C1—N1—C3	95.27 (14)	C17—C16—C21	118.0 (2)
C22—N1—C3	130.58 (16)	C17—C16—C7	123.1 (2)
O2—N2—C5	105.81 (13)	C21—C16—C7	118.9 (2)
O2—N2—C4	110.17 (14)	C16—C17—C18	120.8 (3)
C5—N2—C4	108.50 (13)	C16—C17—H17	119.6
O1—C1—N1	132.72 (18)	C18—C17—H17	119.6
O1—C1—C2	135.53 (18)	C19—C18—C17	120.7 (3)
N1—C1—C2	91.72 (15)	C19—C18—H18	119.7
C4—C2—C1	116.34 (16)	C17—C18—H18	119.7
C4—C2—C3	106.57 (14)	C18—C19—C20	119.5 (3)
C1—C2—C3	85.72 (13)	C18—C19—H19	120.2
C4—C2—H2	114.8	C20—C19—H19	120.2
C1—C2—H2	114.8	C19—C20—C21	120.6 (3)
C3—C2—H2	114.8	C19—C20—H20	119.7
N1—C3—C5	117.72 (15)	C21—C20—H20	119.7
N1—C3—C2	87.24 (14)	C20—C21—C16	120.4 (3)
C5—C3—C2	105.83 (14)	C20—C21—H21	119.8
N1—C3—H3	114.2	C16—C21—H21	119.8
C5—C3—H3	114.2	C23—C22—C27	119.77 (18)
C2—C3—H3	114.2	C23—C22—N1	119.34 (17)
N2—C4—C29	108.99 (15)	C27—C22—N1	120.88 (17)
N2—C4—C2	101.55 (14)	C22—C23—C24	120.83 (19)
C29—C4—C2	114.43 (15)	C22—C23—H23	119.6
N2—C4—H4	110.5	C24—C23—H23	119.6
C29—C4—H4	110.5	C25—C24—C23	119.5 (2)
C2—C4—H4	110.5	C25—C24—H24	120.3
N2—C5—C7	105.14 (15)	C23—C24—H24	120.3
N2—C5—C3	102.61 (14)	O5—C25—C24	124.7 (2)
C7—C5—C3	118.17 (15)	O5—C25—C26	115.79 (19)
N2—C5—H5	110.1	C24—C25—C26	119.50 (19)
C7—C5—H5	110.1	C27—C26—C25	121.1 (2)
C3—C5—H5	110.1	C27—C26—H26	119.5
O2—C6—C15	107.66 (14)	C25—C26—H26	119.5
O2—C6—C8	104.47 (14)	C26—C27—C22	119.4 (2)
C15—C6—C8	108.99 (15)	C26—C27—H27	120.3
O2—C6—C7	106.06 (13)	C22—C27—H27	120.3
C15—C6—C7	114.39 (15)	O5—C28—H28A	109.5
C8—C6—C7	114.54 (14)	O5—C28—H28B	109.5
C16—C7—C5	116.80 (16)	H28A—C28—H28B	109.5
C16—C7—C6	115.20 (15)	O5—C28—H28C	109.5
C5—C7—C6	101.63 (14)	H28A—C28—H28C	109.5
C16—C7—H7	107.5	H28B—C28—H28C	109.5
C5—C7—H7	107.5	C4—C29—Se1	110.03 (13)
C6—C7—H7	107.5	C4—C29—H29A	109.7
O3—C8—C9	122.98 (18)	Se1—C29—H29A	109.7
O3—C8—C6	122.14 (17)	C4—C29—H29B	109.7
C9—C8—C6	114.72 (15)	Se1—C29—H29B	109.7
C14—C9—C10	118.95 (18)	H29A—C29—H29B	108.2
C14—C9—C8	119.98 (18)	C31—C30—C35	118.4 (2)
C10—C9—C8	120.96 (17)	C31—C30—Se1	122.08 (17)
C11—C10—C9	120.5 (2)	C35—C30—Se1	119.48 (17)
C11—C10—H10	119.7	C30—C31—C32	120.1 (2)
C9—C10—H10	119.7	C30—C31—H31	119.9
C10—C11—C12	119.6 (2)	C32—C31—H31	119.9
C10—C11—H11	120.2	C33—C32—C31	120.4 (3)
C12—C11—H11	120.2	C33—C32—H32	119.8
C13—C12—C11	121.4 (2)	C31—C32—H32	119.8
C13—C12—H12	119.3	C34—C33—C32	119.8 (2)
C11—C12—H12	119.3	C34—C33—H33	120.1
C12—C13—C14	119.5 (2)	C32—C33—H33	120.1
C12—C13—H13	120.3	C33—C34—C35	120.9 (2)
C14—C13—H13	120.3	C33—C34—H34	119.6
O4—C14—C9	123.26 (17)	C35—C34—H34	119.6
O4—C14—C13	116.63 (17)	C30—C35—C34	120.3 (2)
C9—C14—C13	120.1 (2)	C30—C35—H35	119.8
O4—C15—C6	112.56 (16)	C34—C35—H35	119.8
			
C6—O2—N2—C5	15.91 (18)	C8—C9—C10—C11	174.9 (2)
C6—O2—N2—C4	−101.18 (16)	C9—C10—C11—C12	0.3 (3)
C22—N1—C1—O1	−6.5 (4)	C10—C11—C12—C13	0.5 (4)
C3—N1—C1—O1	179.8 (2)	C11—C12—C13—C14	−0.3 (4)
C22—N1—C1—C2	175.33 (19)	C15—O4—C14—C9	16.9 (3)
C3—N1—C1—C2	1.69 (14)	C15—O4—C14—C13	−163.62 (19)
O1—C1—C2—C4	−73.3 (3)	C10—C9—C14—O4	−178.98 (18)
N1—C1—C2—C4	104.78 (17)	C8—C9—C14—O4	4.8 (3)
O1—C1—C2—C3	−179.7 (2)	C10—C9—C14—C13	1.5 (3)
N1—C1—C2—C3	−1.60 (13)	C8—C9—C14—C13	−174.72 (19)
C1—N1—C3—C5	−108.13 (18)	C12—C13—C14—O4	179.7 (2)
C22—N1—C3—C5	77.9 (2)	C12—C13—C14—C9	−0.7 (3)
C1—N1—C3—C2	−1.67 (14)	C14—O4—C15—C6	−48.8 (2)
C22—N1—C3—C2	−175.64 (18)	O2—C6—C15—O4	−55.5 (2)
C4—C2—C3—N1	−114.74 (15)	C8—C6—C15—O4	57.3 (2)
C1—C2—C3—N1	1.49 (12)	C7—C6—C15—O4	−173.07 (16)
C4—C2—C3—C5	3.32 (19)	C5—C7—C16—C17	−32.9 (3)
C1—C2—C3—C5	119.55 (15)	C6—C7—C16—C17	86.3 (3)
O2—N2—C4—C29	−84.46 (17)	C5—C7—C16—C21	147.02 (19)
C5—N2—C4—C29	160.14 (15)	C6—C7—C16—C21	−93.8 (2)
O2—N2—C4—C2	154.42 (13)	C21—C16—C17—C18	−0.5 (4)
C5—N2—C4—C2	39.02 (17)	C7—C16—C17—C18	179.5 (2)
C1—C2—C4—N2	−118.02 (17)	C16—C17—C18—C19	0.4 (5)
C3—C2—C4—N2	−24.55 (18)	C17—C18—C19—C20	0.0 (5)
C1—C2—C4—C29	124.75 (19)	C18—C19—C20—C21	−0.3 (5)
C3—C2—C4—C29	−141.78 (17)	C19—C20—C21—C16	0.2 (4)
O2—N2—C5—C7	−31.16 (17)	C17—C16—C21—C20	0.1 (4)
C4—N2—C5—C7	87.05 (16)	C7—C16—C21—C20	−179.8 (2)
O2—N2—C5—C3	−155.33 (14)	C1—N1—C22—C23	−160.4 (2)
C4—N2—C5—C3	−37.13 (18)	C3—N1—C22—C23	11.3 (3)
N1—C3—C5—N2	114.86 (17)	C1—N1—C22—C27	20.8 (3)
C2—C3—C5—N2	19.55 (18)	C3—N1—C22—C27	−167.57 (18)
N1—C3—C5—C7	−0.2 (2)	C27—C22—C23—C24	0.6 (3)
C2—C3—C5—C7	−95.50 (18)	N1—C22—C23—C24	−178.19 (19)
N2—O2—C6—C15	−117.18 (16)	C22—C23—C24—C25	−0.1 (3)
N2—O2—C6—C8	127.06 (14)	C28—O5—C25—C24	−8.2 (3)
N2—O2—C6—C7	5.68 (18)	C28—O5—C25—C26	172.7 (2)
N2—C5—C7—C16	159.75 (15)	C23—C24—C25—O5	−179.9 (2)
C3—C5—C7—C16	−86.6 (2)	C23—C24—C25—C26	−0.8 (3)
N2—C5—C7—C6	33.51 (17)	O5—C25—C26—C27	−179.7 (2)
C3—C5—C7—C6	147.19 (16)	C24—C25—C26—C27	1.1 (3)
O2—C6—C7—C16	−151.09 (16)	C25—C26—C27—C22	−0.6 (3)
C15—C6—C7—C16	−32.6 (2)	C23—C22—C27—C26	−0.3 (3)
C8—C6—C7—C16	94.2 (2)	N1—C22—C27—C26	178.50 (18)
O2—C6—C7—C5	−23.81 (18)	N2—C4—C29—Se1	−174.50 (12)
C15—C6—C7—C5	94.68 (18)	C2—C4—C29—Se1	−61.6 (2)
C8—C6—C7—C5	−138.47 (15)	C30—Se1—C29—C4	175.59 (15)
O2—C6—C8—O3	−96.2 (2)	C29—Se1—C30—C31	−50.9 (2)
C15—C6—C8—O3	148.92 (19)	C29—Se1—C30—C35	129.36 (18)
C7—C6—C8—O3	19.3 (3)	C35—C30—C31—C32	−0.3 (4)
O2—C6—C8—C9	79.28 (18)	Se1—C30—C31—C32	180.0 (2)
C15—C6—C8—C9	−35.6 (2)	C30—C31—C32—C33	1.0 (4)
C7—C6—C8—C9	−165.14 (15)	C31—C32—C33—C34	−1.2 (5)
O3—C8—C9—C14	−178.1 (2)	C32—C33—C34—C35	0.6 (4)
C6—C8—C9—C14	6.5 (2)	C31—C30—C35—C34	−0.2 (3)
O3—C8—C9—C10	5.8 (3)	Se1—C30—C35—C34	179.50 (18)
C6—C8—C9—C10	−169.71 (17)	C33—C34—C35—C30	0.1 (4)
C14—C9—C10—C11	−1.3 (3)		

### Hydrogen-bond geometry (Å, °)

**Table:**

*D*—H···*A*	*D*—H	H···*A*	*D*···*A*	*D*—H···*A*
C5—H5···O1^i^	0.98	2.42	3.199 (3)	136
C29—H29A···O5^ii^	0.97	2.54	3.465 (3)	160

Symmetry codes: (i) −*x*+3/2, *y*−1/2, −*z*+1/2; (ii) *x*+1/2, −*y*+1/2, *z*−1/2.
